# How an agreement with restriction of unhealthy food marketing and sodium taxation influenced high fat, salt or sugar (HFSS) food consumption

**DOI:** 10.1186/s12889-024-18069-w

**Published:** 2024-02-23

**Authors:** Nongnuch Jindarattanaporn, Sirinya Phulkerd, Aphichat Chamratrithirong, Rossarin Soottipong Gray, Umaporn Pattaravanich, Nutnicha Loyfah, Sasinee Thapsuwan, Natjera Thongcharoenchupong

**Affiliations:** Institute for Population and Social Research, Mahidol University, Salaya Campus, 999 Phutthamonthon 4 Road, Phutthamonthon, 73170 Nakhon Pathom, Thailand

**Keywords:** Agreement, Policy, Sodium taxation, Food marketing restriction, Thais

## Abstract

**Background:**

Food taxation and food marketing policy are very cost-effectiveness to improve healthy diets among children. The objective of this study was to investigate the socio-demographic characteristics of Thais and attitude towards on policy unhealthy food marketing restriction and sodium taxation which influence high fat, sodium, and sugar (FHSS) food eating.

**Methods:**

The data were obtained from the 2021 Health Behavior of Population Survey, four-stage sampling method of the Thai people, aged 15 years and above, using a offline survey application-assisted face-to-face interview. Logistic Regression were used to analyze the explanatory variables on agreement and HFSS food intake.

**Results:**

Almost half (48.4%) of samples disagreed with sodium taxation, and 42.7% of the samples disagreed with food marketing restriction. Most (99.6%) of Thai respondents consumed HFSS food, including sugar sweetened beverages (SSB). Gender, age, education, income, BMI, and health status were associated with agreement with food marketing restriction policy and sodium taxation policy. There is no association between agreement with policy on sodium taxation and food marketing and HFSS food consumption.

**Conclusion:**

Nearly half of Thais indicated that they disagreed with policy on food marketing restriction and sodium taxation. Therefore, understanding and awareness of the two policies among Thais should be further investigated in order to develop better policy communication for increased public understanding and engagement.

## Background

At least 2.8 million global deaths annually are attributable to overweight or obesity [1] Unhealthy diet is one of risk factor of overweight, obesity, and for diet-related non-communicable diseases (NCDs) such as diabetes, hypertension, and cardiovascular diseases [2–4] Unhealthy diet refers to food that contains a high level of fat, salt and sugar (HFSS), and lacks nutrients such as fiber, vitamins and minerals [5] According to the Thai National Health Examination Survey in 2021, the prevalence of overweight in people age ≥ 15 years in Thailand was 42.2% (37.8% in males, 46.4% in females), with a significant increase from the level in the survey in 2014, which was 37.5% (32.9% in males, 41.8% in females) [6] The consumption of HFSS contributes to overweight, obesity, and hypertension among Thais age 15 years or above [4, 7, 8]. The prevalence of childhood obesity rose from 5.3% in 1995 to 11.4% in 2014 among children aged 1–5 years and from 5.8 to 13.9% in the same period among children aged 6–14 years [9].

Thais consume more food high in fat, sugar, and salt today than they ever did before. In 2021, high-fat food accounted for the highest percentage of food consumption among Thais age 6 years old or above (88.1%), followed by sugar-sweetened beverages (SSB) (60.4%), instant foods (50.3%), and snacks (44.6%) [10] Furthermore, the trend of unhealthy food intake among Thais continues to worsen. The percentage of HFSS food consumption among Thais increased from 77.4% in 2017 to 88.1% in 2021 for high-fat food, and from 28.5 to 43.9% for Western-style fast food (i.e., international chain outlet food) [10, 11] In addition, Thais consumed salt and sugar at levels above the WHO recommended daily amount. A cross-sectional population-based survey conducted in Thailand in 2021 revealed that average sodium consumption among Thai adults was 3.6 g per day which was more than double the WHO recommended daily amount of salt [12] An average Thai person consumes 25.5 teaspoons of sugar per day, and that is four times more than the WHO recommended limit [13].

There is evidence that restricting a child’s exposure to unhealthy food and beverage (F&B) advertising on TV is one of the most cost-effective interventions for governments to improve obesity-related health outcomes [14–16]. In Thailand, earlier cost-benefit analyses have predicted that implementation of a policy to restrict children’s exposure to unhealthy food advertising on TV would reduce body mass index (BMI) in 6–12 year-olds by an average 0.32 kg/m2 and would cost the government 1.13 million baht to implement in 2019. Such a policy was predicted to lead to a reduction in the prevalence of overweight/obese Thai children by 121,000 cases [17].

Over the years, Thailand has attempted to improve children’s healthy food consumption. In 2021, the Bureau of Nutrition (BON), Department of Health, Ministry of Public Health drafted a law to regulate F&B marketing that affects children’s health. The draft bill aims to reduce the exposure to, power of F&B marketing to children age under 18 years and improve children’s dietary intakes and health outcomes [18]. The draft Act, outlines nine provisions for the restriction of unhealthy food marketing, including: restrictions apply comprehensively across media and settings to restrict any actions to promote, advertise, disseminate or publicise a product or to promote sales. The legislation specifically prohibits the use of premium offers or sales inducements in marketing for unhealthful foods, such as the use of giveaways, price promotions or competitions. The draft legislation also precludes the sale of unhealthful foods at educational establishments and children’s centers, and includes provisions for labelling to signpost unhealthful foods [18].

HFSS food taxation is a potentially cost-effective intervention to address the problem of obesity and NCDs [19, 20] Studies have found that fiscal policy can be effective in altering intake and purchase of targeted F&B [21, 22]. A systematic review studies and a study in Chile confirmed that sodium taxation can reduce sodium consumption [23–26] As an attempt to reduce sodium consumption, Thailand has set ambitious goals to achieve a 30% reduction in population-level intake of salt/sodium [27] and this is in line with the WHO global voluntary targets for a 30% relative reduction in mean population intake of salt/sodium by 2025 (relative to 2010 levels) [28]. Lessons learned from a previous study indicate that increasing SSB tax can reduce SSB consumption, BMI and obesity prevalence in Thailand [21]. As a result, Thailand has been considering the implementation of a sodium tax since 2018 [29]. However, at the time of this research, there was no sign that this policy would become in effect because the Excise Department wanted more time to allow the economy to recover from the adverse economic impact of Covid-19 [30].

Despite the fact that the desired nutrition-friendly laws have not yet been enacted in Thailand, it is still important to assess public opinion regarding pending policies related to unhealthy food and food-related NCD. The information from those assessments can inform the design and implementation of effective and sustainable policies in this domain. Public opinion provides valuable insight into the concerns, needs, and expectations of the general population [31] Public opinion on food policy, food marketing restrictions, and sodium taxation, can affect the viability of said policies, since politicians tend to be more willing to implement a policy if it receives popular support [32]. Support for healthy food policy not only increases when the population is aware of the effects of unhealthy diets and the environmental causes of obesity [33] but also depends on sociodemographic factors (e.g., age, gender, education) which can be more or less favorable toward the implementation of such policies [34, 35]. To the best of our knowledge, no research on public opinion on sodium taxation policy and food marketing restrictions policy overweight/obese and the factors influencing public opinion (on the two policies referenced above) has been conducted. Nor has there been a study of the association between public opinion on sodium taxation policy and food marketing restrictions policy and HFSS food intake in Thailand.

Therefore, the objective of this study was to investigate factors that are associated with agreement with food marketing restrictions policy and sodium taxation policy, and HFSS food consumption among Thais age 15 years or above. This study should provide important findings to guide policy development in tackling obesity and NCD in Thailand, especially regarding the impact of agreement with food marketing restrictions policy and sodium taxation policy on eating behaviors of Thais.

## Methods

This cross-sectional study was conducted using data from the 2021 round of the national Survey of the Health Behavior of the Population [10]. This survey was conducted by the Thai National Statistical Office (NSO).

### Patient and public involvement statement

The interviewers by NSO explained and identified a survey topics and questions of importance to respondents., if the respondents did not understand any questions, they asked to the interviewers to clarify the questions.

### Sampling and sample size

**Fig. 1:**
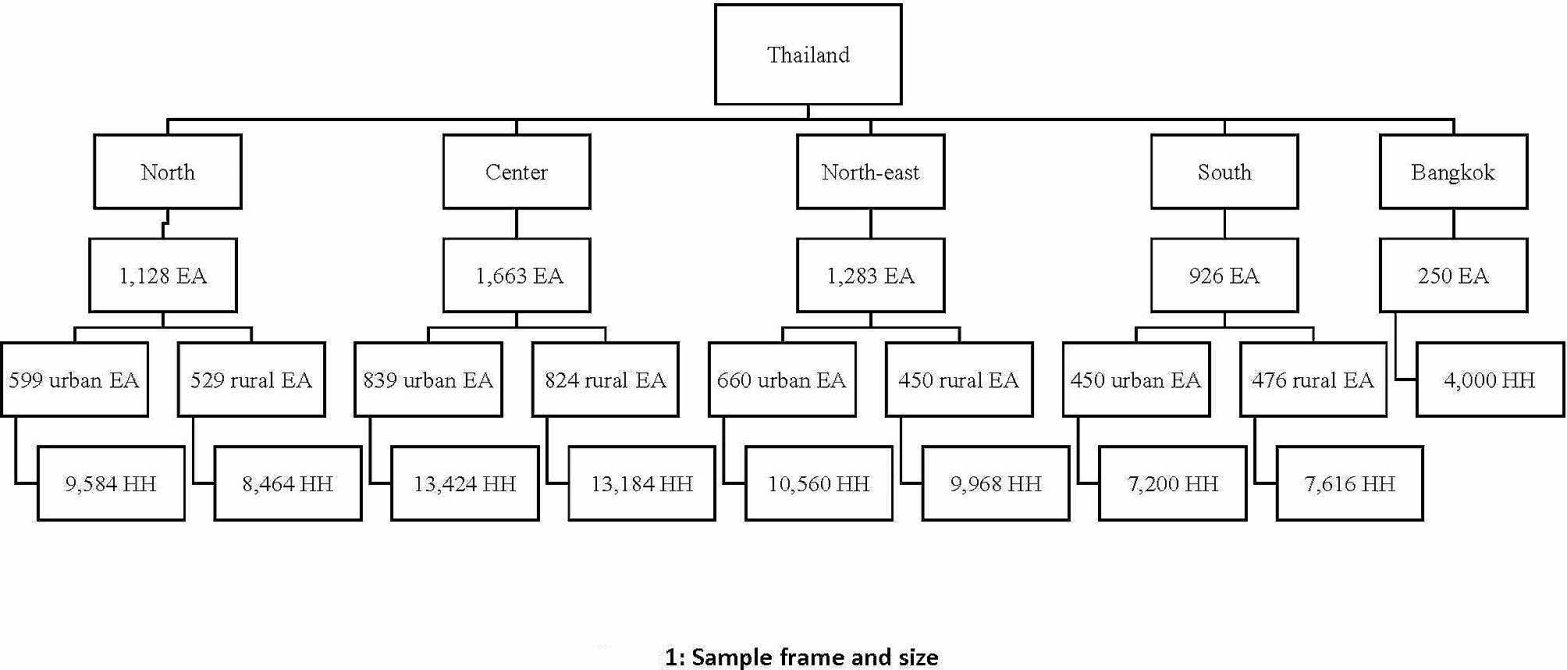
Sample frame and size

A national survey with a multi-stage sampling method was conducted among population age six years or above in Thailand by the NSO. Of the total of 139,088 EAs across Thailand, 5,250 enumeration areas (EAs) were systematically sampled from each region, with 2,798 EAs in urban and 2,452 EAs in rural areas. Next, 84,000 households (HH) were listed and systematically selected by the NSO from each EA, with 44,768 households in urban and 39,232 households in rural areas, for the total of 5,250 EAs. There were 16 households for each EA, and all the household members were asked to take part in the survey. Finally, 73,654 households and 86,216 participants agreed to be interviewed (response rate = 95.2%). This study focused only on Thai citizens age 15 years or above. After attrition, a net total of 86,094 persons were included for analysis in this study (Fig. 1).

### Data collection

Before data collection, a hands-on training workshop was organized with the interviewers by NSO. The workshop aimed to help them understand the study protocol, definition, questionnaire, and assigned tasks, and develop hands-on survey skills. The well-trained staff used an offline survey application to administer the questionnaire on a tablet computer. The data were collected by face-to-face interview using the structured questionnaire. The staff contacted and met the head of household. The data were collected from all the household members who were present. If the data collector failed to reach a household member after three visits, that member was excluded; a replacement was not taken. Before each interview, the head of household and household members were asked for permission to participate in the survey. Data were collected from February 1 to May 30, 2021.

**Fig. 2:**
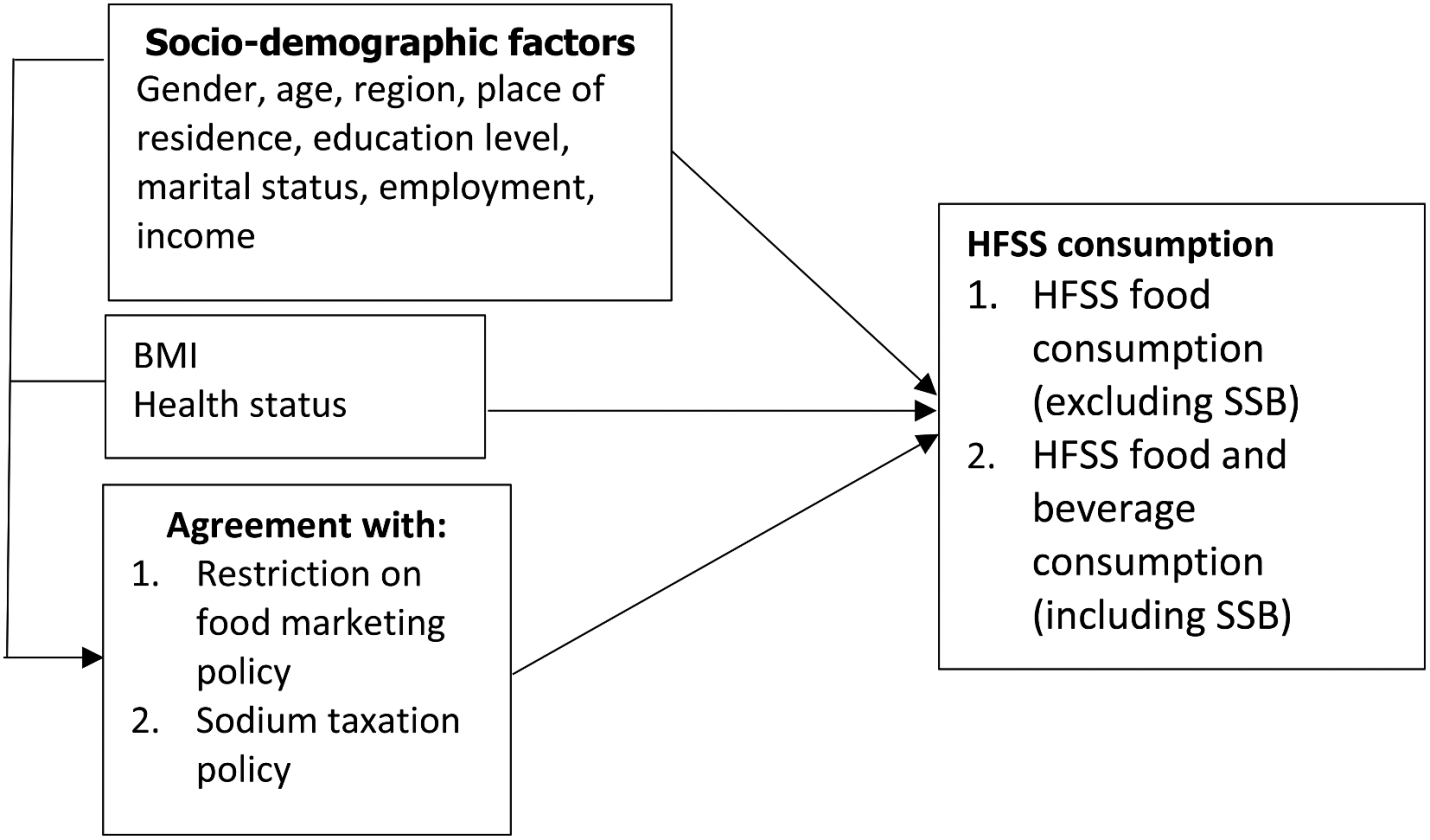
Conceptual framework

Figure 2 illustrates the conceptual framework of this study. This conceptual framework was developed and derived from the findings of the literature review [36, 37]. Previous evidences illustrated that there were certain sociodemographic characteristics and health-related behaviours factors (e.g., gender, age, education level, region of residence, marital status, employment status, personal health concerns, etc.) which might significantly impact agreement with sodium taxation policy and restriction of food marketing policy [36, 37]. Therefore, this study took into account individual factors on sociodemographic characteristics, as well as response to policy change through policy agreement of individual [38].

### Measurements

#### Dependent variables

Consumption of foods with high fat, sodium, and sugar (HFSS) was asked of sample respondents using a single question: ‘*In the last month, how often did you consume each of the following food groups: frozen foods, high-fat foods, Western fast food, instant foods, desserts, sugar-sweetened beverages, and freshly-made beverages?”* Frequency of consumption was categorized into six groups: (1) every day, (2) 5 to 6 days per week, (3) 3 to 4 days per week, (4) 1 to 2 days per week, (5) 1–3 days per month, and (6) no consumption. HFSS in this study refers to food & drink products that are high in (saturated) fat, sodium or sugar according to the Department of Principles of Food and Nutrition for Health [39].

Then HFSS consumption was recoded into two variables: HFSS food consumption (excluding SSB) and HFSS F&B consumption (including SSB). HFSS food consumption was computed on a composite scale that combined ‘*yes*’ and ‘*no’* answers in the five food variables ranging from 0 to 5. Then, we grouped these responses into two categories: 0 = no, and 1–5 = yes. HFSS F&B consumption was computed on a composite scale that combined ‘*yes*’ and ‘*no’* answers in the seven food variables ranging from 0–7. Then, we grouped these responses into two categories: 0= ‘*no*,’ and 1–7= ‘*yes*.’ Accordingly, these two variables were categorical variables. HFSS consumption was categorised as this calculation because there was low frequency of each category of each food groups. Therefore, we recoded HFSS consumption into two groups.

#### Policy agreement variables

Participants were asked whether they agreed or disagreed if Thailand implemented the following government policies: (1) Restriction of unhealthy food marketing; and (2) Sodium taxation. The response is considered to be key information to inform policy development to combat obesity and NCDs [10] Response was measured on a Likert scale ranging from zero to two, where 0 = “*I’m not sure;*” 1= “*I disagree;”* and 2 = “*I agree.*” We grouped these responses into two categories: “*not sure*” and “*disagree*” (0); and “*agree*” (1). In addition, agreement with sodium taxation policy and restriction food marketing policy were dependent variables when we examined the association between socio-demographic characteristics and agreement with the two policies.

#### Socio-demographic characteristics variables

Gender was included as a dichotomous variable; i.e., male or female. Age was categorized into three groups: 15–24, 25–59, and 60 or above, to distinguish between life stages such as adolescence to adulthood, working age, and retirement years. Socio-demographic characteristics of respondents included regional area of residence (Bangkok, Central, North, South, Northeast), place of residence (urban/rural), educational level, marital status (single/ married/ windowed/ divorced/ separated), employment (employee/ unemployed), monthly income (< 15,000/ 15,001–30,000/ >30,000 baht), and health status (has/hasn’t a chronic illness). The criteria of the International Obesity Task Force (IOTF) were used to classify and describe overweight and obesity among the sample [40]. BMI was categorized into three groups: <18.5, 18.5–22.9, and ≥ 23.0, to distinguish between underweight, normal weight, and overweight/obese. Ethnicity, though usually viewed as an important factor in studies of other populations, was not included as one of the confounders because nearly all (97.3%) members of the Thai population are ethnic Thais.

### Data analysis

Weighted analysis, considering a complex survey design, was performed to estimate frequencies and percentages of the socio-demographic characteristics of the sample, their agreement with food marketing restrictions and sodium taxation, and their HFSS consumption. Binary logistic regression models were used to determine socio-demographic factors which were statistically associated with agreement with sodium taxation and food marketing restriction, and associated with the independent variables (i.e., agreement with sodium taxation and food marketing restriction) and HFSS consumption of the sample. Records with missing data were excluded case-wise when tabulating frequencies and modelling outcomes. SPSS version 18 was used for all analyses. Variables with a p-value < 0.05 were considered statistically significant.

## Results

### Summary of participant characteristics on sociodemographic status, HFSS food consumption and agreement with policies

Table 1 shows the socio-demographic characteristics of the sample of 86,094 respondents. The sample comprised 56.8% female and 43.2% male. The mean (± standard deviation, sd) age of the study subjects was 48.9 (± 16.6) years with a range of 15 to 103 years. Half the sample lived in a rural area. Overall, more than half of respondents disagreed with the policy on food marketing restrictions and sodium taxation. The majority of respondents had no NCD or chronic condition at the time of the interview, and half were overweight/obese. A higher proportion of respondents who had attained a bachelor’s degree or higher education agreed with food marketing restrictions and sodium taxation compared to those who were illiterate. A higher proportion of respondents with monthly income > 30,000 baht agreed with food marketing restrictions and sodium taxation than those who earned < 15,000 baht.

**Table 1:** Socio-demographic characteristics, health and nutritional status of Thais by opinion of two nutrition-friendly policies in 2021

Socio-demographic characteristics	n	Agreement with food marketing restriction		Agreement with sodium taxation	
		Disagree; not sure (n = 54,684)	Agree (n = 31,410)	Disagree; not sure (n = 58,496)	Agree (n = 27,598)
Overall	86,094	63.5	36.5	67.9	32.1
**Gender**					
Male	37,174	64.1	35.9	68.5	31.5
Female	48,920	63.0	37.0	67.6	32.4
**GenderAge (years) (median = 50, mean = 48.9, sd = 16.6, max = 103, min = 15)**					
15–24	7,289	65.6	34.4	69.6	30.4
25–59	54,405	62.4	37.6	66.8	33.2
≥ 60	24,399	65.5	34.5	69.9	30.1
**Regional area of residence**					
Bangkok	11,056	60.3	39.7	66.1	33.9
Central	26,175	69.0	31.0	72.5	27.5
North	15,157	63.9	36.1	67.8	32.2
Northeast	22,510	62.7	37.3	68.2	31.8
South	11,195	55.0	45.0	59.0	41.0
**Place of residence**					
Urban	40,141	65.8	34.2	67.2	32.9
Rural	45,952	62.8	37.4	68.6	31.4
**Educational level (n = 86,021)**					
Illiterate	3,435	71.8	28.2	75.3	24.7
Primary or lower	40,132	66.3	33.7	70.9	29.1
Secondary	23,019	63.2	36.8	67.9	32.1
Vocational or college	7,426	60.9	39.1	64.9	35.1
Bachelor or higher	12,008	54.2	45.9	57.9	42.1
**Marital status (n = 86,081)**					
Single	17,712	62.5	37.5	66.6	30.4
Married	52,724	62.8	37.2	67.4	32.6
Windowed	9,992	68.8	31.2	73.1	26.9
Divorced/ separated	5,652	64.2	35.8	68.6	31.4
**Employment**					
Unemployed	25,011	65.8	34.2	69.9	30.2
Employed	61,083	62.6	37.4	67.2	32.8
Income (baht/month) (median = 6,850, mean = 9,802, sd = 11,247, max = 99,998, min = 100)					
< 15,000	71,124	64.9	36.1	69.5	30.5
15,001–30,000	11,106	58.2	41.8	62.1	37.9
> 30,000	3,864	52.3	47.7	56.1	43.9
BMI (median = 23, mean = 23.5, sd = 3.9, max = 53.2, min = 10.8) (n = 82,730)					
Underweight (< 18.5)	5317	64.9	35.1	70.4	29.6
Normal weight (18.5–22.9)	35,556	62.6	37.4	66.7	33.3
Overweight/obese (≥ 23.0)	41,323	63.7	36.3	68.5	31.4
Health status					
No NCD or chronic condition	61,342	63.4	36.6	67.7	32.3
Have an NCD or chronic condition	24,752	63.9	36.1	68.5	31.5

*Note* n = samples of this study

As shown in Table 2, nearly all (99.6%) respondents consumed HFSS food, including SSB. Four-fifths of the sample reported they ate high-fat foods (86.7%), followed by more than half who drank freshly-made beverages (59.6%), and SSB (57.8%). Whereas, two-fifths (45.7%) of respondents consumed instant foods, followed by Western fast food (38.8%), and desserts (35.4%). For food frequency, one-fourth (26.1%) of Thais drank freshly made beverages every day, while one-third (30.9%) consumed high-fat foods, with a frequency of 1–2 days per week.

**Table 2:** HFSS food consumption in 2021

Variables	(n = 86,094)	
	Frequency	Percentage
**Frozen foods (n = 85,919)**		
No	57,018	66.4
Yes	28,901	33.6
**High-fat foods (n = 85,981)**		
No	11,460	13.3
Yes	74,520	86.7
**Western fast food (franchise) (n = 85,968)**		
No	52,631	61.2
Yes	33,337	38.8
**Instant foods (n = 85,999)**		
No	46,713	54.3
Yes	39,286	45.7
**Desserts (n = 85,931)**		
No	55,497	64.6
Yes	30,434	35.4
**Sugar sweetened beverages (SSB) (n = 85,967)**		
No	36,262	42.2
Yes	49,705	57.8
**Freshly made beverages (n = 85,944)**		
No	34,727	40.4
Yes	51,216	59.6
**HFSS food consumption (excluding SSB) (n = 85,711)**		
No	6,327	7.4
Yes	79,383	92.6
**HFSS F&B consumption (n = 85,591)**		
No	3,409	0.4
Yes	82,121	99.6

*Note* n = samples of this study

### Association between sociodemographic characteristics and policy agreement

Table 3 shows the associations between sample characteristics and agreement policy on sodium taxation and food marketing restrictions. Female participants were more likely to support food marketing restrictions (AOR = 1.092, 95%CI: 1.059–1.125), and sodium taxation (AOR = 1.084, 95%CI: 1.051–1.118), compared with males. Respondents age 25–59 years and 60 years or above agreed with food marketing restrictions and sodium taxation, compared with those age 15–24 years. Respondents in higher education groups were more likely to support food marketing restrictions and sodium taxation, compared to the illiterate. Respondents in high- and moderate-income groups were more likely to agree with food marketing restrictions and sodium taxation compared with those in the lower-income group. In addition to regional area, compared to respondents living in Bangkok, those living in South and Central were more likely to support sodium taxation and food marketing restrictions. Respondents who were normal weight were more likely to agree with food marketing restrictions and sodium taxation compared with those who were underweight. Thais who had a chronic health condition were more likely to support food marketing restrictions and sodium taxation, compared with those had no NCD or chronic condition.

**Table 3:** Factors associated with agreement with food marketing restrictions policy and sodium taxation policy in 2021

Factors	Agreement with food marketing restrictions (n = 86,094)				Agreement with sodium taxation (n = 86,094)			
	AOR	Sig.	95% CI		AOR	Sig.	95% CI	
			Lower Bound	Upper Bound			Lower Bound	Upper Bound
**Gender**								
Male	1	-			1	-	-	-
Female	1.092*	**0.000**	**1.059**	**1.125**	1.084*	**0.000**	**1.051**	**1.118**
**Age** (years)								
15–24	1	-			1	-	-	-
25–59	1.115*	**0.001**	**1.048**	**1.186**	1.106*	**0.002**	**1.038**	**1.179**
≥ 60	1.148*	**0.000**	**1.070**	**1.232**	1.139*	**0.000**	**1.059**	**1.224**
**Education**								
Illiterate	1	-	-	-	1	-	-	-
Primary or lower	1.240*	**0.000**	**1.143**	**1.346**	1.250*	**0.000**	**1.147**	**1.362**
Secondary	1.456*	**0.000**	**1.337**	**1.585**	1.466*	**0.000**	**1.341**	**1.603**
Vocational or college	1.593*	**0.000**	**1.450**	**1.750**	1.633*	**0.000**	**1.481**	**1.802**
Bachelor or higher	1.835*	**0.000**	**1.674**	**2.013**	1.930*	**0.000**	**1.753**	**2.124**
**Regional area**								
Bangkok	1	-			1	-	-	-
Central	0.696*	**0.000**	**0.662**	**0.732**	0.776*	**0.000**	**0.738**	**0.820**
North	0.934*	**0.017**	**0.883**	**0.988**	1.045	0.140	0.986	1.107
Northeast	1.014	0.600	0.962	1.0670	1.062*	**0.034**	**1.005**	1.122
South	1.301*	**0.000**	**1.226**	**1.381**	1.444*	**0.000**	**1.359**	**1.535**
**Place of residence**								
Urban	1	-	-	-	1	-	-	-
Rural	1.023	0.170	0.990	1.057	1.007	0.668	0.975	1.042
**Marital status**								
Single	1	-			1	-	-	-
Married	0.993	0.729	0.952	1.035	0.991	0.674	0.949	1.034
Windowed	0.816*	**0.000**	**0.765**	**0.870**	0.814*	**0.000**	**0.761**	**0.870**
Divorced/ separated	0.965	0.300	0.902	1.032	0.964	0.305	0.900	1.034
**Employment**								
Unemployed	1	-	-	-	1	-	-	-
Employed	1.070*	**0.000**	**1.0321**	**1.111**	1.038	0.061	0.998	1.079
**Incomes**								
< 15,000 (low)	1	-			1	-	-	-
15,001–30,000 (moderate)	1.176*	**0.000**	**1.122**	**1.233**	1.204*	**0.000**	**1.147**	**1.264**
> 30,000 (high)	1.379*	**0.000**	**1.278**	**1.487**	1.445*	**0.000**	**1.339**	**1.560**
**BMI**								
Underweight (< 18.5)	1	-	-	-	1	-	-	-
Normal weight (18.5–22.9)	1.079*	**0.015**	**1.015**	**1.147**	1.164*	**0.000**	**1.091**	**1.240**
Overweight and obese (≥ 23.0)	1.023	0.460	0.962	1.089	1.060	0.075	0. 994	1.131
**Health status**								
No disease	1	-	-	-	1	-	-	-
Having chronic health condition	1.107*	**0.000**	**1.067**	**1.148**	1.095*	**0.000**	**1.054**	**1.137**

*Note* n = samples of this study; * indicates when *p-*value is < 0.05; AOR = adjusted odds ratio; Sig = Statistical significance level; 95% CI = 95% confidence interval

### Association between policy agreement and HFSS F&B consumption

Table 4 shows agreement with food marketing restrictions and sodium taxation was associated with HFSS consumption. Socio-demographics characteristics of samples were introduced into the model. The binary logistic regression analysis found that there was no statistically-significant association between agreement with food marketing policy sodium taxation policy and HFSS food consumption after controlling gender, age, education, region of residence, place of residence, marital status, employment, income, and BMI with HFSS consumption, including SSB.

**Table 4:** Association between agreement with food marketing restrictions policy and salt taxation policy and HFSS intake in 2021

Factors	HFSS F&B consumption (including SSB) (n = 85,591)				HFSS food consumption (excluding SSB) (n = 85,711)			
	AOR	Sig.	95% CI		AOR	Sig.	95% CI	
			Lower Bound	Upper Bound			Lower Bound	Upper Bound
**Gender**								
Male	1	-	-	-	1	-	-	-
Female	0.783*	**0.000**	**0.723**	**0.846**	1.093*	**0.002**	**1.032**	**1.157**
**Age** (years)								
15–24	4.853*	**0.000**	3.717	6.336	4.618*	**0.000**	**3.818**	**5.587**
25–59	1.999*	**0.000**	1.815	2.202	1.817*	**0.000**	**1.692**	**1.951**
≥ 60	1	-	-	-	1	-	-	-
**Education**								
Illiterate	1	-	-	-	1	-	-	-
Primary or lower	1.140*	**0.093**	**0.979**	**1.327**	1.200*	**0.003**	**1.063**	**1.356**
Secondary	2.082*	**0.000**	**1.729**	**2.507**	1.808*	**0.000**	**1.574**	**2.076**
Vocational or college	2.082*	**0.000**	**1.631**	**2.659**	1.853*	**0.000**	**1.563**	**2.195**
Bachelor or higher	2.709*	**0.000**	**2.133**	**3.440**	2.096*	**0.000**	**1.781**	**2.467**
**Regional area**								
Bangkok	1	-	-	-	1	-	-	-
Central	1.427*	**0.000**	**1.207**	**1.688**	1.451*	**0.000**	**1.304**	**1.614**
North	0.923	0.354	0.779	1.093	1.223*	**0.001**	**1.091**	1.371
Northeast	0.548*	**0.000**	**0.468**	**0.643**	0.692*	**0.000**	**0.624**	**0.769**
South	1.001	0.989	0.832	1.205	1.054	0.399	0.933	1.190
**Place of residence**								
Urban	0.751*	**0.000**	**0.690**	**0.818**	0.871*	**0.000**	**0.819**	**0.927**
Rural	1	-	-	-	1	-	-	-
**Marital status**								
Single	1.054	0.632	0.850	1.306	1.239*	**0.003**	**1.075**	1.429
Married	0.890	0.183	0.750	1.057	0.994	0.921	0.886	1.116
Windowed	0.731*	**0.001**	**0.608**	**0.879**	0.838*	**0.008**	**0.735**	**0.955**
Divorced/ separated	1	-	-	-	1	-	-	-
**Employment**								
Unemployed	1	-	-	-	1	-	-	-
Employed	1.791*	**0.000**	**1.644**	**1.951**	1.430*	**0.000**	**1.339**	**1.527**
**Income** (monthly, baht)								
< 15,000 (low)	1.180	0.224	0.904	1.540	1.427*	**0.000**	**1.220**	**1.670**
15,001–30,000 (moderate)	1.688	0.001	1.253	2.273	1.410*	**0.000**	**1.194**	**1.665**
> 30,000 (high)	1	-	-	-	1	-	-	-
**BMI**								
Underweight (< 18.5)	1	-	-	-	1	-	-	-
Normal weight (18.5–22.9)	1.095	0.189	0.956	1.254	0.998	0.966	0.894	1.113
Overweight and obese (≥ 23.0)	1.415*	**0.000**	**1.233**	**1.624**	1.157*	**0.010**	**1.036**	**1.292**
**Health status**								
No NCD or chronic condition	1	-	-	-	1	-	-	-
Have NCD/chronic condition	1.046	0.287	0.963	1.137	1.060	0.073	0.995	1.129
**Agreement with food marketing restriction**								
Not sure and disagree	1.049	0.228	0.971	1.133	-	-	-	-
Agree	1	-	-	-	-	-	-	-
**Agreement with sodium taxation**								
Not sure and disagree	-	-	-	-	0.995	0.861	0.938	1.055
Agree	-	-	-	-	1	-	-	-

*Note* n = samples of this study; * indicates when *p-*value is < 0.05; AOR = adjusted odds ratio; Sig = Statistical significance level; 95% CI = 95% confidence interval

## Discussion

This study examined factors to identify statistical associations with agreement with sodium taxation policy and food marketing restrictions policy, and factors influencing policies on HFSS consumption. Thais in this national sample disagreed with policy on food marketing restrictions and sodium taxation, and a high percentage regularly consume HFSS food. After hypothesis testing, the results indicate that the factors influencing agreement with food marketing restrictions policy and sodium taxation policy are as follows: gender, age, education, regional area of residence, place of residence, marital status, income, BMI, and health status. The analysis found no association between agreement with the two policies and HFSS food intake, but agreement with food marketing restrictions policy and sodium taxation policy was found to be significantly associated with socio-demographic characteristics, including BMI.

Almost half the sample disagreed with policy on food marketing restrictions. According to public relations concept, information dissemination is intended to inform citizens to maintain a positive or favorable view about an entity, its leadership, products, or political decisions. That said, poor public relations may result in a lack of understanding and support for the host entity or its political decisions [41]. Therefore, a further study is needed to investigate Thai people’s understanding and awareness of the two policies. This finding is inconsistent with evidence from a cross-European survey to explain the level of public support for different types of healthy eating policy in Europe. That study found that the highest percentage in favor of banning HFSS food advertising targeted at children was 85.4% in Spain, followed by Italy (70.8%), the UK (67.1%), and Belgium (66.1%) [37]. The reason why Thais disagreed with the food marketing policy might be due to the dominant narrative, backed by mass media [42, 43] that eating habits are, above all, a matter of individual choice and responsibility [44]. Even so, scientific evidence indicates that the main determinants of diet are social and environmental factors [45, 46]. Thus, findings from the present study may be useful for policymakers and public health advocates seeking publicly-acceptable policy on sodium taxation and food marketing restrictions as solutions for obesity prevention. Some researchers have sought to identify the most persuasive means such as sharing obesity problem and narratives emphasizing societal causes and solutions for obesity to communicate the evidence for regulations in order to improve support for regulations [48, 49].

More than half of this national sample of Thais disagreed with sodium tax policy. This finding is consistent with studies in Ireland, Belgium, Denmark, Italy, Poland, and the UK which found less support for taxing high-salt food [36, 37]. By contrast, they had greater resistance to proposed increases in the cost of unhealthy foods, and that may be attributable to the fact that people are likely to be differentially affected by such a tax [50].

In the present study, female participants were more likely to support sodium taxation and food marketing restrictions compared with their male counterparts. This finding is consistent with studies in Australia, Ireland, and Spain which found that women showed greater support for policy on sodium taxation and food marketing restrictions than men [36, 43, 51]. This might be due to the fact that, in recent years, there has been growing concern among consumers about the nutritional quality of the products they consume, with this phenomenon being more marked among women, who are more deeply involved in following good dietary habits and maintaining healthy lifestyles [34, 35, 52]. In addition, women were most likely to support the regulations because they believed them likely to be effective in encouraging healthy eating and reducing population obesity [51].

Respondents in this study age 25–59 and 60 + years agreed more with food marketing restrictions compared with the youngest cohort, i.e., those age 15–24 years. This may be due to the fact that adolescents are more exposed to HFSS marketing strategies and may not think critically about the information as to whether the product is a healthy choice or not [53]. Some evidence revealed that food industry use celebrity that young people love as a presenter to promote their product [54]. This can make them feel connected to the marketed food product business, building trust and relationships. It can lead to disagreement of young people with food marketing policy. Thais with higher educational attainment were more likely to support sodium taxation and food marketing restrictions compared to the illiterate. That findings is consistent with a study of adults in the USA which found that those with less than a college education had 1·7 to 2·6 times the odds of being neutral or opposed to said policies [55]. Presumably, those with higher education were more health conscious and, thus, more supportive of nutrition-friendly policies [56].

In this study, respondents with high or moderate income were more likely to agree with food marketing restrictions policy compared with those in the lower-income group. This finding is consistent with a study in Australia which found that people who had higher socio-economic status (SES) were significantly more likely than those with lower SES to support restrictions of unhealthy food advertising on TV. Only lower-SES participants were less likely to support restrictions on sports sponsorship of unhealthy food and drink advertising, and that may reflect an increased sensitivity to the impact of such regulation on the ability to raise funds for children’s sporting activities [50]. The agreement with food marketing restrictions policy among people depends on the activities of food marketing. Therefore, if the Thai government would like to control sponsorship, e.g., in the school setting, it would be important to provide short-term alternative funding sources to the school (e.g., via the Thai Health Promotion Foundation) to ensure their viability while they transitioned to alternative funding sources.

Sample respondents in high- and moderate-income groups were more likely to agree with sodium taxation policy compared with those in the lower-income group. The results of this study confirm the microeconomic theory that demand is generally considered to slope downward: At higher prices, consumers buy less or the increase in price will discourage customers from buying a product [57]. This finding is consistent with studies in Australia and Ireland which found that people who had lower income opposed a tax on high-salt foods [36, 51]. Taxes may increase financial stress for those who already have lower income, without addressing other influences on food choices. People who had lower income were the most disadvantaged group, and they were less likely than any other group (higher- and moderate-income groups) to increase their support for taxes if the revenue raised was used to subsidize healthy foods. This suggests that products targeted by taxes are consumed for reasons beyond low cost, and may maintain their appeal even when price is adjusted relative to healthier options [51, 58].

The analysis found no association between agreement with policy on food marketing and taxation and HFSS food consumption. This may be due to the fact that changing one’s eating behavior is a slow and long-lasting process [59]. Thus, F&B companies increasingly market directly to the youngest generation to instill habits early [60, 61]. Policy to restrict food marketing to children can effectively reduce children’s exposure to food marketing and its persuasive techniques, and may also reduce their purchases of unhealthy foods [61]. Food marketing restrictions and sodium taxation are policies to support healthy eating behavior, and the aim of these policies is to manage environmental factors (price and marketing of unhealthy products) that influence eating behavior, especially among youth [59, 62]. In addition, the F&B industry sets prices in order to influence consumer acceptance of the product [63]. For sodium taxation, fiscal measures and price controls can reduce demand for unhealthy products by making them more expensive, and thus less appealing to the average consumer [26, 64]. Therefore, changing unhealthy eating behaviors does not necessarily relate to attitudes because the environment in which people develop their dietary behaviors and make their food choices may be a more significant influence on what they eat [62].

This study was conducted using a cross-sectional design, and factors influencing public opinion on food marketing restrictions and sodium taxation policy and policy and HFSS food consumption can be explained within the study period. Therefore, the findings cannot be used to conclude cause-and-effect relationships. Asking respondents about agreement on the two policies may be halo effect [65] because respondents agreed with the policies to bring themselves into favor with the interviewees. They might desire to eat HFSS food. Therefore, there was no association between agreement with food marketing restrictions and sodium taxation and HFSS consumption. Asking respondents about eating behaviors in the past month may be vulnerable to recall bias. Some respondents did not answer the question clearly if they had trouble recalling what they did one month in the past. However, the data was collected using face-to-face interviews, and that may have reduced recall bias when compared to response to mail-in questionnaires or by electronic platforms [66]. Asking participants about opinion on policy on food marketing restrictions did not separate the techniques of marketing. Therefore, future studies should identify or classify marketing techniques in order to evaluate public acceptability of these techniques. Other policies that influence obesity (e.g., food availability, food labelling) were not included in this study. Assessing approval of broader obesity policy initiatives should be the focus of future research in this area.

## Conclusions

This study found that more than half of a national sample of Thais disagreed with food marketing restrictions and sodium taxation for the prevention of obesity. That said, agreement with food marketing policy and sodium taxation policy did not influence HFSS food consumption. This is perhaps due to the fact that there has been limited public relations to improve understanding of the consequences of unhealthy food choices. In addition, there might be considerable misunderstanding or confusion about the two policies with regard to their impact on overweight/obesity. Therefore, the Ministry of Public Health should communicate the evidence that is the basis for policy on food marketing and sodium taxation in order to improve national support for these two polices and to minimize deleterious unintended consequences.

## Data Availability

The datasets generated and/or analysed during the current study are not publicly available due these data were used under license of the Thai NSO but are available from the corresponding author on reasonable request and with the permission of The Thai NSO.
